# Genome-Scale Technology Driven Advances to Research into Normal and Malignant Haematopoiesis

**DOI:** 10.6064/2012/437956

**Published:** 2012-12-31

**Authors:** Berthold Göttgens

**Affiliations:** Department of Haematology, Cambridge Institute for Medical Research, Cambridge University and Wellcome Trust and MRC Stem Cell Institute, Hills Road, Cambridge CB2 0XY, UK

## Abstract

Haematopoiesis or blood development has long served as a model system for adult stem cell biology. Moreover, when combined, the various cancers of the blood represent one of the commonest human malignancies. Large numbers of researchers have therefore dedicated their scientific careers to studying haematopoiesis for more than a century. Throughout this period, many new technologies have first been applied towards the study of blood cells, and the research fields of normal and malignant haematopoiesis have also been some of the earliest adopters of genome-scale technologies. This has resulted in significant new insights with implications ranging from basic biological mechanisms to patient diagnosis and prognosis and also produced lessons likely to be relevant for many other areas of biomedical research. This paper discusses the current state of play for a range of genome-scale applications within haemopoiesis research, including gene expression profiling, ChIP-sequencing, genomewide association analysis, and cancer genome sequencing. A concluding outlook section explores likely future areas of progress as well as potential technological and educational bottlenecks.

## 1. Introduction

Haematopoiesis represents the process whereby multipotential blood stem and progenitor cells differentiate into more than 10 distinct mature blood cell types. Research over the last 30 years has led to the development of purification protocols that permit the isolation of many of these progenitor and all mature cell types at close to 100% purity. Moreover, biological assays have been developed to validate the functional properties for most of these different cell types including the many progenitors at various stages of maturity. Consequently, differentiation of the blood system is better defined than any other mammalian organ system and has hence become a model system for the wider field of stem cell biology.

Since many of the mature blood cell types are short lived, they need to be constantly replenished throughout adult life, with the consequence that the blood system has one of the fastest turnovers of all human organ systems. Production of the various types of mature blood cells is tightly controlled, with transcription factor and signalling proteins playing particularly prominent roles [1–5]. Long-term formation of mature blood cells from blood stem cells also forms the basis of successful bone marrow transplantation, which therefore represents one of the most widely used stem cell treatments currently in use. Transplantation of blood stem cells has also been used as a powerful assay when applied to experimental animals, in particular rodents. Here it allows for the detection of the presence of blood stem cells in complex mixtures of cells, with the most advanced protocols allowing for the transplantation of a single blood stem cell to give rise to long-term donor-derived haematopoiesis in the transplant recipient [6].

The various types of human leukaemias all share the property of perturbed blood cell production, often with an accumulation of the so-called blast cells that resemble immature blood progenitor cells [7]. With transcription factor and signalling genes being key to normal blood development, it is perhaps no surprise that acquired mutations in these categories of genes are now recognised as one of the commonest causes of leukaemia development [8–11]. Below I will outline how a range of genome-scale approaches has been employed to provide significant advances to our understanding of both normal and malignant haematopoiesis. This will be followed by a brief outlook on likely future developments and relevance beyond the field of haematopoiesis research.

## 2. Gene Expression Profiling for Network Inference and Disease Classification

The relative ease of accessing blood cells compared with most other human tissues likely is a major reason as to why several advanced approaches for the extraction of new biological knowledge from large-scale gene expression profiling datasets have been pioneered in studies using blood cells. Below I focus in particular on gene expression profiling studies aimed at regulatory network reconstruction and disease classification.

With the ever-increasing momentum of genome-scale science driven by, for example, human genome project [12, 13], gene expression profiling has rapidly been recognised as a powerful means to define the phenotype of a given cell population. With differentiation not only entailing but most likely being driven by changes in gene expression profiles, generating gene expression profiles for a range of different but related cell types has the potential to identify those aspects of a given expression profile that are characteristic for a given cell type. Moreover, large-scale analysis across multiple different cell types and lineages can be used to define coexpressed gene clusters, which through the use of reverse-engineering approaches can be utilised further for the reconstruction of likely regulatory hierarchies and networks. An early example of this approach was the development of the ARACNE (algorithm for the reconstruction of accurate cellular networks algorithm [14]). In this study, the authors reported the reconstruction of regulatory networks from expression profiles of human B cells, which suggested the existence of hierarchical, scale-free networks, where a few highly interconnected hub genes account for most of the interactions. The authors also identified the MYC protein as a major hub controlling a network of known and previously unknown MYC target genes, some of which represented major hubs themselves. This and related approaches have subsequently been used for the analysis of normal and pathologic networks in a range of mammalian cells [15–24].

Coexpression across more extended differentiation hierarchies has more recently been utilised for the definition of haematopoietic gene sets as well as the inference of regulatory networks [25]. Here the authors generated gene expression profiles for 38 distinct purified human hematopoietic cell populations. Subsequent use of probabilistic models and analysis of *cis*-elements were employed to further define the regulatory circuitry, which lead to the definition of densely interconnected *cis*-regulatory circuits and a number of transcription factors where their differential expression across the different hematopoietic lineages was inferred to be involved in the generation of distinct cellular states. Together with earlier human studies [26] as well as comprehensive expression profiling in the mouse [27–30], this study provides a rich resource for hypothesis generation for future research into the molecular controls of blood cell differentiation.

Gene expression profiling has also been employed widely across a range of different haematological malignancies, in order to identify new classification schemes with potential diagnostic, prognostic, and/or therapeutic value. One of the early protagonists in the field has been the group of Louis Staudt, who in 2000 reported distinct types of diffuse large B-cell lymphoma through the use of gene expression profiling [31]. Diffuse large B-cell lymphoma represents the most common subtype of non-Hodgkin's lymphoma and was known to be clinically heterogeneous. Using extensive expression profiling, the authors identified two molecularly distinct disease forms with gene expression patterns indicative of different stages of B-cell maturation, and differential overall survival. Similar studies have subsequently been performed on a wide range of leukaemias [32–49] and more recently also at very large scale in other malignancies [50]. Molecular classification of tumours on the basis of gene expression therefore has the capacity to identify previously undetected and clinically significant subtypes of cancer.

## 3. Gene Expression Profiling Compendia as Resources for Hypothesis Generation

With the ever-increasing availability of expression profiling datasets, efforts have been initiated to collate expression data from diverse studies into unified data collections with a view to providing powerful data mining platforms. One example is represented by BloodExpress, an expression profiling resource for mouse haematopoiesis [28]. Through a user-friendly web interface, BloodExpress allows for searching of uniformly processed microarray datasets. BloodExpress covers both mature and progenitor populations and indeed includes expression data for the majority of all mouse blood cell types. Identification of dynamic gene expression changes is therefore facilitated, as expression data can be retrieved for a variety of differentiation pathways within the well-defined haematopoietic differentiation tree. Both gene-centric and cell-type centric interfaces are implemented, with the latter also allowing for filtering by specific gene functional categories, and thus further facilitating the use of BloodExpress for building novel hypotheses [51–53].

While BloodExpress demonstrated the feasibility of integrating gene expression profiles from diverse laboratories, cross-dataset normalisation did represent a formidable challenge, so that much of the expression information was eventually discretised in a binary “on/off” fashion, thus giving some confidence to expression states but also losing potentially vital information in quantitative expression changes. Parallel efforts have therefore been initiated to generate unified expression profiling resources generated by a single laboratory or facility. One particularly prominent example here is the hematopoietic fingerprints database from the Goodell lab [27]. The hematopoietic fingerprints database contains expression profiles for hematopoietic stem cells as well as their differentiated progeny, such as granulocytes, erythrocytes, natural killer cells, monocytes, activated, and naive T cells and also B cells. The database can be downloaded or accessed through the web, or even through a smartphone application. For their publication, the authors also used the database to generate novel hypotheses on the transcriptional control of the NK cell lineage and the monocyte lineage, where they were able through functional experiments to implicate the transcription factors Zfp105 and Ets1, respectively, in the differentiation of these two lineages. These results together with many subsequent citations [54–59] of their paper proved the extraordinary utility of this resource.

Comparable expression profiling compendia have more recently also been generated for the human haematopoietic system. The first such study generated a web-accessible resource termed the HaemAtlas [26]. This resource contains gene expression profiles generated from a single centre that cover human erythroblasts, megakaryocytes, B cells, cytotoxic and helper T cells, natural killer cells, granulocytes, and monocytes. Bioinformatic analysis focussing on specific functional categories reported cell type specific characteristics related to transcription factor genes as well as immunoglobulin superfamily members. As the above resources, the HaemAtlas is freely accessible and has therefore played an important role in accelerating hypotheses generation, particularly within the context of genomewide association studies [60–65]. A more extended human expression profiling resource was published subsequently by the lab of Ben Ebert [25]. A total of 38 different human progenitor and mature blood populations are contained within this resource, which also has a web portal known as DMap or differentiation map portal. The free accessibility of this resource again has meant that it has been rapidly used by the community [66–69]. Moreover, a compendium of gene expression profiles for malignant blood cells has also recently been reported [70], which again through a user-intuitive website facilitate data analysis and hypothesis generation. Extensive analysis and visualization tools allow for the integrated analysis of more than 5,800 leukaemia and normal haematopoiesis samples, with easy data retrieval obviating the need for potentially redundant investigations.

A particularly exciting recent development has been an attempt to generate an expression profiling compendia that will allow for an absolute expression quantification [29]. Here more than 10,000 different gene expression profiles were integrated to explore the previously unknown and variable sensitivities of each probe set. The resulting Gene Expression Commons database utilises statistical attributes of each microarray probe (e.g., dynamic range and also threshold) to define absolute expression levels for each gene. The web-based platform represents an implementation with 39 highly purified mouse blood stem/progenitor/differentiated populations and covers almost all of the mouse haematopoietic system. The software is implemented as an open platform, so that individual investigators cannot only explore expression levels of genes or gene families, but also upload their own datasets into the database for cross-comparisons. It is likely that this approach will not only contribute to a very significant progress in haematopoiesis research, but also be applied to many other biomedical research fields, since it appears to overcome some of the previous limitations inherent to cross-comparisons of microarray datasets.

## 4. Epigenomic Analysis

While a universally accepted definition of epigenetics as well as epigenomics has this far eluded the field, the current consensus view is that modification to the DNA sequence (e.g., methylation) as well as posttranslational modification to chromatin proteins (e.g., histone modifications) represents key aspects of the epigenetic control of gene regulation. Following the advent of high-throughput sequencing technologies, it was quickly realised that genomewide analysis of histone modification status is particularly amenable to this new technology. To this end, the sheared chromatin fragments are subjected to sequencing following immunoprecipitation with suitable antibodies (chromatin immunoprecipitation or ChIP), with the whole technique commonly referred to as ChIP-Seq. Posttranslational modifications indicating both active and repressed transcriptional status are well recognized and have been mapped at genome scale in a variety of both mouse and human blood cell types [71–80].

It is hoped that genome-scale chromatin maps will eventually provide information complementary to gene expression profiling, with implication for human disease prediction, diagnosis, prognosis, and treatment. Indeed, the European Union invested close to 30 million euro in 2011 into its new BLUEPRINT initiative [81]. BLUEPRINT brings together 41 leading European universities, research institutes, and industry entrepreneurs, with the primary goal of mapping chromatin status in human blood cells from healthy and diseased individuals and to provide at least 100 reference epigenomes to the scientific community. Resource-generating activity will be complemented by research into blood-based diseases, including common leukaemias and autoimmune disease (Type 1 Diabetes). The recurring theme therefore is that once again a new ground-breaking initiative uses blood cells as their experimental model of choice.

Genome-scale information on DNA methylation can be obtained using a variety of approaches, with direct sequencing of the entire genomic DNA following bisulfite treatment perhaps representing the most comprehensive approach. Indeed, dramatic improvements and falling costs of high throughput sequencing have made bisulfite sequencing (BS-Seq) a viable option for the global analysis of DNA methylation [82–86]. The well-understood nature of the haematopoietic differentiation tree has again made application of these technologies to blood cells the model of choice for studying the nature of dynamic changes in DNA methylation [83, 87, 88]. Lastly, recent studies demonstrated that ten-eleven translocation (Tet) proteins can catalyze 5methyl-cytosine (5mC) oxidation and generate 5mC derivatives, including 5-hydroxymethylcytosine (5hmC). Importantly, Tet family proteins and 5hmC appear to play key roles in normal development as well as in many diseases, with mutations in human leukaemia patients representing a particularly prominent example [89–103]. Concerted research efforts are currently directed therefore at generating genomewide maps of both the locations of 5hmC as well as the binding sites of Tet proteins [96, 97, 104–110].

## 5. Genome-Scale Transcription Factor Maps

When using antibodies that specifically recognise transcription factor proteins, the ChIP-Seq technology is also readily amenable to determining genomewide transcription factor binding maps. Indeed, one of the earliest reports using this technology mapped the locations of the NRSF transcriptional regulator in a T-lymphoid cell line [111]. Transcription factor ChIP-Seq studies have now been performed for more than 30 different factors across all major branches of the haematopoietic differentiation tree, as well as a range of mouse and human leukaemia cells and cell lines [51, 68, 76, 78, 79, 112–123]. Important lessons that have been learned from these studies include that (1) high confidence transcription factor binding events highlight functional gene regulatory sequences [117], (2) multifactor studies can reveal previously unrecognised combinatorial interactions between pairs or groups of TFs [118], (3) combinatorial binding events can be used to localise genes that are candidate regulators of developmental processes such as blood cell differentiation [78], and (4) studies from different laboratories can be readily integrated and exploited to perform bioinformatic searches across large numbers of different studies [113]. 

Transcription factor ChIP-Seq maps have also been utilised to map the genomewide locations of leukaemogenic factors. One recent example mapped the locations of the RUNX1/ETO fusion protein, which is a leukaemia-initiating transcription factor that interferes with RUNX1 function [124]. Global analysis of chromatin status, transcription factor binding, and gene expression showed that RUNX1/ETO controls important regulators of hematopoietic differentiation and self-renewal. Moreover, the removal of RUNX1/ETO re-established the RUNX1 binding profile seen in normal blood cells and also caused the inhibition of leukemic proliferation and self-renewal, thus highlighting the potential therapeutic value of targeting abnormal transcriptional processes in cancer. Other studies have mapped the genomewide locations of the PML-RAR leukaemia oncogene [77, 125], as well as mutant Notch1 [126], which is recognised as one the most commonly mutated genes in T-cell leukaemia [127]. Collectively, these studies have already demonstrated that application of ChIP-Seq technology to the study of transcription factor oncogenes has the potential to provide new mechanistic insights with potential therapeutic value.

## 6. Genomewide Association Studies

Genomewide association studies (GWASs) examine many common genetic variants in different individuals to see if any variant is associated with a particular trait. GWAS surveys typically focus on associations between single-nucleotide polymorphisms (SNPs) and traits for major diseases, but can also identify variants that are linked to phenotypic variation seen in normal populations. Blood parameters such as the numbers of a particular blood cell type per millilitre of blood vary between different individuals, and much of this variation is thought to be genetic. Since elevated or reduced levels of certain blood cell types can predispose to diseases, GWAS studies have been performed to identify some of the underlying variants. In particular, blood platelets play a pivotal role in cardiovascular disease through their involvement in atherothrombosis following coronary artery plaque rupture. Not only does the platelet response to such an event vary between individuals, but also this variation is largely genetically controlled. A recent integrated systems biology approach performed high density genotyping of 110 genes in a cohort of more than 500 individuals with known levels of platelet response, which was followed by gene expression profiling and proteomics studies on platelets from individuals with the so-called “extreme end” response phenotypes [128]. With the advent of high density (i.e., 500,000 SNPs) genotyping arrays, a large number of case and control samples can now be tested at an affordable cost. By making use of the recently completed Wellcome Trust Case Control Consortium (WTCCC) study, common sequence variants that are associated with abnormal blood parameters and/or confer risk to myocardial infarction have been identified. Total white blood cell count and its constituent subtypes were recently used to study several 10,000 subjects [129, 130], to determine genetic factors influencing variability within the blood parameters. Ten variants associated with total white blood cell count were identified with additional variants associated with variation in specific blood cell types. Possible functional relationships between the relevant candidate genes were predicted by integrating gene expression and pathways-based analyses, which revealed functional connectivity among implicated loci.

GWAS studies are commonly followed up by meta-analysis of several independent studies. Analysis of 66,867 individuals of European ancestry followed by extensive biological and functional assessment identified 68 loci reliably associated with platelet count and volume [130]. Expression profiling demonstrated a tendency for lineage specific expression within the haematopoietic differentiation tree for the genes identified by GWAS. Using a functional followup in zebrafish and drosophila, 11 of these genes were validated as novel regulators of blood cell formation, thus providing an example of successful translation of GWAS studies to generating new functional insights.

## 7. Cancer Genome Sequencing

Identification of the whole complement of genes that are mutated in human cancers and therefore drive malignant transformation has been a central aim of cancer research ever since the advent of recombinant DNA technology. Following the development of ultra-high-throughput sequencing technologies and the subsequent reductions in the costs of sequencing whole genomes, projects have been initiated in Europe, America, and Asia to decode entire genomes from multiple individuals for a wide range of human cancers. Identification of somatically acquired mutations is thought to provide the most direct route towards delineating genes critical to the development of human cancers, yet it is not entirely clear at this stage what will be the most reliable techniques to distinguish these so-called “driver” mutations from the so-called “passenger” mutations; that are also found in the tumour but occurred by chance without providing a selective advantage to tumour growth and/or survival.

Within haematopoiesis research, cancer genome sequencing has now been applied to several different haematological malignancies [69, 131–145]. For example, multiple myeloma which is an incurable malignancy of plasma cells, was investigated by the sequencing of 38 tumour genomes and their comparison to matched normal DNAs, which revealed several new and unexpected putative oncogenic mechanisms [146]. These included mutations of genes involved in protein translation, histone methylation, and blood coagulation, thus demonstrating that cancer genome sequencing of large collections of samples can yield new and previously unsuspected insights into cancer.

A more recent study investigating cancer genomes in patients with acute myeloid leukaemia (AML) specifically addressed the question of passenger and driver mutations [147]. Of note, normal karyotypes are common and genomic instability is unusual in AML. By comparing AML samples with a known initiating event (PML-RARA) with normal karyotype AML samples and nonleukaemic blood stem/progenitor cells, this study suggests that most mutations in AML genomes are random events that occurred prior to the initiating leukaemogenic mutation, and that in many cases, only one or two additional, cooperating mutations are needed to generate the malignant founding clone. Cells from the founding clone can acquire additional cooperating mutations, yielding subclones that can contribute to disease progression and/or relapse, thus making clonal analysis of cancers an important goal of current research efforts. Indeed, it was again an analysis using blood cells as a model which provided an important recent breakthrough. The particular study in question reported a method for sequencing complete exomes from single cells [148]. The authors went on to use this method to perform whole-exome single-cell sequencing for 58 single cells from a patient with myeloproliferative neoplasm. This analysis suggested that in this particular patient, the neoplasm followed the pathway of monoclonal evolution. Importantly, this technological breakthrough now sets the scene for similar analyses in other human leukaemias as well as solid cancers.

## 8. Outlook

The application of new genome-scale technologies has resulted in an unprecedented increase in the amounts of data available for hypothesis generation in biomedical research. However, the extent to which this explosion in data has contributed to an actual increase in mechanistic understanding has so far been less impressive. To some extent this is to be expected, since the new datasets have been available at most for a few years, and mechanistic studies often have longer timescales. However, there are also cultural, training/educational, and technical issues that need to be overcome to accelerate the exploitation of new datasets.

In terms of research culture, it has become apparent that many investigators have found a comfortable niche for themselves, where essentially they end up with repeating the same data collection exercise every couple of years, each time with the next version of whatever genome-scale technology they have become an expert in (an example here would be remapping SNPs when the next version SNP array becomes available, or performing ChIP-on-chip analysis one year and follow this with ChIP-Seq of more or less the same samples the next years). This however avoids asking the much harder question whether it is possible to decipher the meaning of any underlying biology from a genome-scale observation and how this might be exploited to deliver actual biomedical advances. Journals and funding agencies should pay more attention to this issue and provide better rewards to those investigators willing to tackle the harder mechanistic questions. Just because an experiment has cost millions of dollars and used the latest technology, this on its own, does not mean that it will have a lasting value.

The educational issue is that the exploitation of genome-scale datasets requires biologists with a robust understanding of bioinformatics and statistics and ideally also a significant knowledge of computer programming languages. Universities are increasingly offering relevant courses, yet the demand for bioinformatically trained biologists is still an outstripping supply. Moreover, the longer-term career path for such scientists is not clear either, at least in academic settings, because of the tensions between the need to performing primary, intellectual novel research in order to achieve academic promotions, which does not blend well with carrying out a bioinformatic support function.

Finally, there are also scientific conceptual issues that impede interpretation and thus exploitation of genome-scale datasets. For example, it is clear that cell fate decisions are made by individual cells, and that indeed there is substantial heterogeneity within a given biological cell population [149–152]. Genome-scale technologies however often require the need to generate material from many thousands to millions of cells and thus can only report population averages. Some recent progress has been achieved at the level of single-cell RNA sequencing and exome sequencing [148, 153–155]. However, data will need to be generated for 100s if not 1,000s of single cells to make sure that the full heterogeneity of a given population has been sampled. Another important issue is that the genome is not a linear sequence, but instead organised in a complex 3-dimensional fashion [156–158]. Chromatin maps as well as transcription factor maps and gene expression data therefore need to be integrated with information on the 3-dimensional structure of the genome. Importantly, there appears to be a rapid progress in our ability to comprehensively map chromosome conformation at genomic scales [159–163]. However, it is likely that many long-range chromosome interactions are not particularly rigid and may indeed be rather transient. Measurements generated from cell populations therefore likely reflect the ensemble of common interactions and will therefore need to be deconvoluted so as to obtain not just one possible “solution” but instead a number of possible 3-D interaction maps. And as with many of the other techniques covered in this paper, the data generation phase is of a descriptive rather than functional nature, so that the mere description of chromosome loops will not provide direct proof of functional relevance.

Given the rapid pace of recent technological innovations particularly in terms of generating descriptive data (the various genome-scale maps), the major roadblocks will be to enhance the throughput of downstream functional studies. Importantly, many of the technologies developed to generate the largely descriptive genome-scale maps can also be adapted to multiplex and thus accelerate the analysis of downstream functional assays. For example, high-throughput next generation sequencing can be adapted to many biological assays that require counting. Reporter gene assays can be replaced by measuring transcript abundance rather than luciferase or lacZ enzyme activity, and through the inclusion of sequence tags, many different promoters can be assayed simultaneously [164]. Another exciting area of recent progress concerns the application of transcription activator-like effector nucleases (TALENs) to perform highly efficient modifications of the genomes, and thus generates all sorts of mutant alleles both in cell lines and also in vivo [165, 166]. The likelihood therefore is that the next decade will not only bring an ever increased rate of generating descriptive whole-genome datasets, but also show substantial acceleration in the generation of new biological insights.
